# Morphological variation of the deciduous second molars in the Baka Pygmies

**DOI:** 10.1038/s41598-021-95524-3

**Published:** 2021-08-13

**Authors:** Petra G. Šimková, Gerhard W. Weber, Fernando V. Ramirez Rozzi, Lotfi Slimani, Jérémy Sadoine, Cinzia Fornai

**Affiliations:** 1 Department of Evolutionary Anthropology, University of Vienna, Vienna, Austria; 2 Core Facility for Micro-Computed Tomography, University of Vienna, Vienna, Austria; 3 UMR7206 Ecoanthropologie, MNHN, CNRS, UP, Musée de L’Homme, Paris, France; 4 EA 2496 Pathologies, Imagerie et biothérapies oro-faciales, Université Paris Descartes, Montrouge, France; 5 UR2496 - Plateforme Imageries du Vivant, Université de Paris, Montrouge, France; 6 Institute of Evolutionary Medicine, University of Zurich, Zurich, Switzerland; 7 Vienna School of Interdisciplinary Dentistry, Klosterneuburg, Austria

**Keywords:** Anthropology, Biological anthropology, Social anthropology, Dentine

## Abstract

The Baka Pygmies are known for their short stature resulting from a reduced growth rate during infancy. They are peculiar also for their teeth erupt earlier than in any other African population, and their posterior dentition is larger than in non-Pygmy populations. However, the Baka’s dental morphology, like several other aspects of their biology, is still understudied. Here, we explore the variation of the Baka’s deciduous upper and lower second molars (dm2s) in comparison to a geographically heterogeneous human sample by means of 3D geometric morphometrics and analysis of dental traits. Our results show that the different populations largely overlap based on the shape of their dm2s, especially the lower ones. Their distal region and the height of the dentinal crown differ the most, with the Baka showing the most extreme range of variation. Upper and lower dm2s covary to a great extent (RV = 0.82). The Baka’s and South Americans’ dm2s were confirmed among the largest in our sample. Despite the Baka’s unique growth pattern, long-lasting isolation, and extreme dental variation, it is not possible to distinguish them from other populations based on their dm2s’ morphology only.

## Introduction

The Baka people are a Central African population with an average male height under 155 cm^1^. Like many other Central African human groups, the Baka have chosen to refer to themselves as Pygmies^2^, accordingly, we will use the term Pygmy in this manuscript for the reasons detailed in Methods.

Until recently, most African Pygmies maintained semi-nomadic lifestyles based mainly on hunting and gathering^1,3^. From the mid-twentieth century, the missionary and sedentarization programs influenced the lifestyle of the Baka populations^4–9^ which moved closer to village sites inhabited by Bantu-speaking agriculturists. Some of the Baka started practicing horticulture in particular seasons of the year and trade products with their neighbors^5,7,8^. Nowadays, the Baka’s hunting camps in the rainforest last only for a few months per year during the major dry season^5,8^, but they remain an essential source of wild resources the Baka are highly depending on^7^.

Short average stature in Pygmy populations results from their particular growth patterns, but the actual underlying evolutionary mechanisms have been long debated^10,11^. Pygmies’ small body size was mainly interpreted as an adaptation to environmental conditions, food shortage^11–13^, and thermoregulation^14^. It is now established that the genetic basis of this specific phenotype is polygenic, including genes related to skeletal growth, allometric patterning, immunity, and metabolism, with effects on the growth hormone axis, in particular the expression of the growth hormone-insulin-like growth factor I^15–17^.

The life history variables in the Baka Pygmies seem to correspond with those of other non-Pygmy populations, including puberty growth spurts and maturity reached at a very similar age^10^. Furthermore, their birth size and birth weight are within the standard limits of modern humans, differently from other Pygmy populations that are already born with smaller body size^6,10^. However, during the first two years of life, the Baka experience a significant deceleration of growth which causes a long-lasting growth delay throughout their whole development and results in a short adult stature (average women’s height = 146.7 cm; average men’s height = 153.5 cm)^10^. Although the timing of tooth eruption is commonly strongly correlated with other life history variables, in the Baka Pygmies the permanent dentition erupts earlier than in any other African groups, including other Pygmy populations (in females upper and lower first molars erupt respectively 0.75 and 0.71 years earlier, while in males 0.63 and 0.39 years earlier)^3^. The Baka’s permanent dentition has been studied with respect to other non-Pygmy populations as well as in relation to their body size^3,7,18–20^. Their post-canine dentition was found to be larger than in their Bantu neighbors^18^ and other non-Pygmy populations. Sexual dimorphism of the Baka’s permanent molars has been identified, being male molars significantly larger than in females^18^. However, no consistent pattern of correlation was found between the Baka’s dental size and body height or weight^19^.

Several important aspects of the Baka’s biology are still unknown, and the morphological variation of the Baka’s teeth is yet unexplored. Given the peculiar set of phenotypical expressions shown by the Baka, we want to contribute to the understanding of the Baka’s morphological variation by investigating their deciduous upper and lower second molars (dm2) compared to a geographically diverse dm2 sample (Table 1). For this purpose, we use two approaches: 3D landmark- and semilandmark-based methods and analysis of qualitative dental traits. Working with µCT-derived 3D surface models, the geometry of both the enamel-dentine junction (EDJ) and the outer enamel surface^21^ can be safely captured, while the roots cannot be investigated since they are completely resorbed in our Baka sample. Similarly, the moderate degree of wear of the occlusal aspect of the crowns permits the investigation of a limited number of discrete dental traits. Based on their results, Sardi and Ramirez Rozzi^22^ inferred that the genetic pathways regulating cranial growth and dental morphogenesis are most likely distinct. If this is the case, we can expect that a reduced growth rate during infancy, resulting in short adult stature in the Baka Pygmies, will not necessarily correspond to dental changes. Thus, we predict that the Baka’s dental shape does not differ substantially from other human groups. Additionally, considering the outcomes of previous work^18^, we expect to find sex-related size differences within the Baka’s dm2s.

**Table 1:** List of deciduous second molars used in this study, with associated information such as repository, sex, degree of wear, and analyses performed. It is also reported whether the dentine horns were virtually reconstructed.

*Population*	*Nr*	*Sex*	*Wear*^23^		*Dental outline analyses*		*EDJ analysis*		*EDJ reconstruction*	
			*U*	*L*	*U*	*L*	*U*	*L*	*U*	*L*
*Baka*^a^	01	f		3		+		+		+
	02	f	3	3	+	+	+	+	+	+
	03	f	3–4		+		+		+	
	04	f	3	3–4	+	+	+	+	+	+
	05	m	3		+		+		+	
	06	f	3–4	3	+	+	+	+	+	+
	08	f	3	3	+	+	+	+	+	+
	09	f		3		+		+		+
	10	f	3	3	+	+	+	+	+	+
	11	m	3		+		+		+	
	12	f	3		+		+		+	
	13	f	3	3	+	+	+	+	+	+
	14	f		3		+		+		+
	16	m	3–4		+		+		+	
	17	m	3–4	3–4	+	+	+	+	+	+
	18	m	3	4–5	+	-	+	+	+	-
	19	f		3		+		+		+
	20	f	3–4	3–4	+	+	+	+	+	+
	22	f		3		+		+		+
	23	m	3	3	+	+	+	+	+	+
	24	f		3–4		+		+		+
	25	m	3	3	+	+	+	+	+	+
	28	m	3		+		+		+	
	29	f		3		+		+		+
	30	m		3		+		+		+
	31	f		3–4		+		+		+
	32	f		3		+		+		+
	33	f		3		+		+		+
	34	m	3	3–4	+	+	+	+	+	+
	35	m	5	3	-	+	+	+	-	+
	36	m		3		+		+		+
	37	m		3		+		+		+
	38	m	3	3	+	+	+	+	+	+
	39	m	3	3	+	+	+	+	+	+
	40	m	3	2	+	+	+	+	+	+
	41	f		3		+		+		+
	43	m	2		+		+		+	
	44	m		5		-		+		-
	45	m		5		-		+		-
*Europeans*	44^b^	?		1		-		+		+
	52^b^	?		1		-		+		+
	57^b^	?		1		-		+		+
	75^b^	?		1		-		+		+
	93^b^	?		1		-		+		+
	96^b^	?		1		-		+		+
	105^b^	?		1		-		+		+
	113^b^	?		1		-		+		+
	115^b^	?		1		-		+		+
	116^b^	?		1		-		+		+
	319^b^	?	1		-		+		+	
	322^b^	?	1		-		+		+	
	429^b^	?		1		-		+		+
	513^b^	?	1		-		+		+	
	549^b^	?	1		-		+		+	
	Nr75^b^	?	1		-		+		+	
	Nr115^b^	?	1		-		+		+	
	Nr116^b^	?	1		-		+		+	
	Nr396^b^	?	1		-		+		+	
	Cs13^c^	?	2		-		+		+	
	Cs305^c^	?	2		-		+		+	
	Cs444^c^	?	1		-		+		+	
	EH-U21^d^	?	1		-		+		+	
	EH-U56^d^	?	1		-		+		+	
	EH-U57^d^	?	1		-		+		+	
	CA_T19^e^	?	1		-		+		+	
	Med1_Batch1^e^	?	1		-		+		+	
	Guid_T49^e^	?	1		-		+		+	
	PM^e^	?	1		-		+		+	
	Tb36^e^	?	1		-		+		+	
	Tb37^e^	?	1		-		+		+	
	Tb44R^e^	?	1		-		+		+	
	Tb49^e^	?	1		-		+		+	
*Bedouins*^f^	BLZ_004	?		3		+		+		+
	BLZ_273	?		3		+		+		+
	BLZ_279	?		1		-		+		+
	BLZ_294	?		1		-		+		+
	BLZ_441	?	1		-		+		+	
	RCEH036	?	2		-		+		+	
*South Americans*^g^	A5380	?	3	2	+	-	+	+	+	+
	A5381	?	3	3	+	+	+	+	+	+
*Egyptians*^g^	A113	?	4–5		-		+		-	
	C120	?	3	3	+	+	+	+	+	+
	C392	m	4	2–3	+	+	+	+	+	+
	CN101	?		2		-		+		+
	CN141	?		3		+		+		+
	CN17	?	3		+		+		+	
	CN233	?		2		-		+		+
	CN26	?	3	3	+	+	+	+	+	+
	CN61	?	3	3	+	+	+	+	+	+
*Southeast Asians*^g^	SI3256	?	1		-		+		+	
	FI3528	?	2	3	-	+	+	+	+	+
	I9664	?	1	1	-	-	+	+	+	+
	I9665	?	4–5		-		+		-	
	NZ_3108	?		3		+		+		+
	NZ_3125-11	?	3		-		+		+	

U = upper; L = lower; f = female; m = male; +  = yes; − = no; ? = unknown; EDJ = enamel-dentine junction.

^a^Université de Paris, Plateforme Imageries du Vivant.

^b^Anatomy Collection of the Medical University of Vienna.

^c^University of Vienna, Department of Evolutionary Anthropology.

^d^Université de Poitiers, Centre de Microtomographie.

^e^University of Bologna, Department of the Cultural Heritage.

^f^Tel Aviv University, Department of Anatomy and Anthropology, The Sackler Maculty of Medicine.

^g^Natural History Museum, Vienna.

## Results

**Figure 1:**
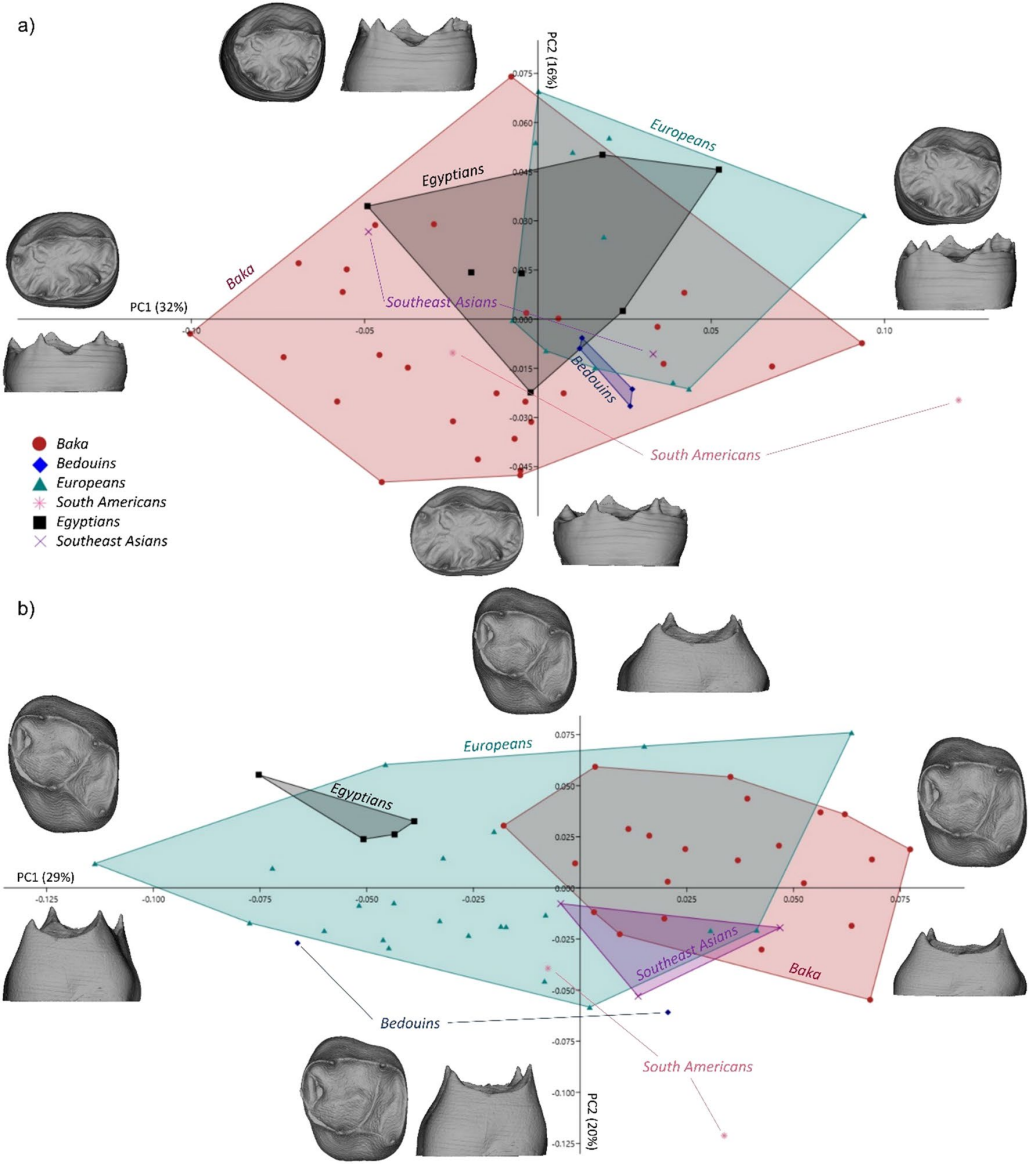
PCA plots for deciduous lower and upper second molar (ldm2 and udm2, respectively) dentinal crowns. (**a**) PC1—PC2 plot for ldm2s in shape space for the combined enamel-dentine junction (EDJ) and cervical outline (warpings at values ± 0.15/ ± 0.07). The Baka show a broader range of morphological variation than the Europeans; **b)** PC1—PC2 plot for udm2s in shape space for the combined EDJ and cervical outline (warpings at values ± 0.15). The Baka show a less variable morphology, while the Europeans exceed the range of variation.

The most salient finding originating from our geometric morphometric investigation of upper and lower dm2s can be summarized as follows. The populations showed an overlapping distribution (Fig. 1; Fig. S1, S2, S3, S4). The dm2s varied from low-crowned and broad to tall and narrow. This pattern of variation was concordant between the upper and lower dm2s. Additionally, a reversed pattern of variation was detected for the upper and lower dm2s: the Baka’s lower dm2s (ldm2s) showed a wider range of variation in comparison to the upper dm2s (udm2s), while the opposite was found for the European upper and lower dm2s. Depending on the analysis, 58 to 71% of the total variance could be explained by the first three principal components (PCs; Table 2).

**Table 2:** Percentage of variance explained by the first three PCs as resulting from the analysis of the deciduous upper and lower second molars’ (udm2s and ldm2s, respectively) cervical outline, crown outline and combined enameldentine junction and cervical outline (dentinal crown).

		PC1 (%)	PC2 (%)	PC3 (%)	Total (PC1-PC3) (%)
**ldm2**	**Dentinal crown**	32	16	10	58
	**Cervical outline**	30	26	15	71
	**Crown outline**	32	21	12	65
**udm2**	**Dentinal crown**	29	20	9	58
	**Cervical outline**	34	23	12	69
	**Crown outline**	37	18	12	67

### Lower dm2 shape variation

The results of the ldm2’s dentinal crowns analysis (using landmarks resampling the EDJ marginal edge and the cervical outline; Fig. 1a), revealed a broad overlap of all populations with the Baka sample. Shape variation along PC1 (explaining 32% of the total variance) concerned the relative bucco-lingual position of the hypoconulid corresponding with the relative bucco-lingual distal expansion of the dentinal crown, as well as its height. Variation along PC2 (16%) was driven by the mesio-distal elongation of the dentinal crown. Both along PC1 and PC2, the Baka exceeded the full range of shape variation within this geographically diverse comparative sample for their particularly short and mesio-distally elongated dentinal crowns, and relatively bucco-lingually narrow distal aspect. We observed a 20% higher variance for the Baka sample (0.006) than for the European ldm2s (0.005) (Supplementary Table 1 online). Supplementary Figure S1 shows the sample distribution along PC3. The Baka male and female means did not differ significantly (*P* = 0.758) in terms of ldm2 dentinal crown shape. Sexual dimorphism could not be tested in the other populations since the individuals’ sex was unknown. As expected, the analysis of the EDJ alone did not provide any additional information with respect to the analysis of the dentinal crown, therefore, we found reporting on this analysis redundant.

The ldm2 cervical outline (see Supplementary Fig. S2a online) varied mainly between round and hourglass-shaped, with the Baka encompassing almost the full range of variation of the considered sample, along PC1 (30%). Along PC2 (26%), the cervical outlines varied from elongated and narrow, to short and broad. The Baka and the South American ldm2s reached the most extreme configurations in terms of bucco-lingual constriction and mesio-distal elongation. Similar to the cervical outline, the crown outline (see Supplementary Fig. S2b online) varied from mesio-distally elongated and bucco-lingually constricted, to rounded along PC1 (32%), while along PC2 (21%), variation occurred in the buccal aspect, with differing relative expansion of the mesial angle with respect to the distal angle.

### Upper dm2 shape variation

The results for the udm2s dentinal crowns (Fig. 1b) showed again an overlapping distribution of Europeans and Southeast Asians with the Baka. Along PC1 (29%), the udm2 dentinal crowns varied between short with squared occlusal aspects, and taller with lingually displaced distal cusps (i.e., metacone and hypocone). Along PC2 (20%), variation was driven by the relative expansion of the trigon (consisting of the three main cusps and forming the central fovea) with respect to the talon (consisting of the disto-lingual cusp and fossa). In other words, the dentinal crowns possessed variably expanded central fovea with respect to their base. The Baka tended to have low, square-shaped dentinal crowns, while Europeans are more variable and are characterized also by tall teeth with lingually shifted distal cusps and reduced metacone. A clear separation between the Baka and the Egyptians, Bedouins, and South Americans was found, although these groups were represented by only two to four specimens. The analysis of variance (see Supplementary Table 1 online) revealed a 40% higher variance in the Europeans’ udm2s (0.007) with respect to the Baka (0.005). Shape variation along PC3 is shown in Supplementary Figure S3 online. The Baka male and female means did not differ significantly (*P* = 0.456) in terms of ldm2 dentinal crown shape. The other groups could not be tested because the information on the individuals’ sex was missing. Similar to the ldm2s, the EDJ analysis for the udm2s was not a source of additional information with respect to the analysis of the dentinal crown.

For the cervical outline (see Supplementary Fig. S4a online), PC1 (34%) reflected the relative expansion of the disto-buccal cusp (or metacone) with respect to the disto-lingual cusp (or hypocone), while, along PC2 (23%), the cervical outlines varied from mesio-distally constricted (hourglass-shaped) to mesio-distally expanded with reduced hypocone. The crown outlines changed from oval to rounded along PC1 (37%; see Supplementary Fig. S4b online). This shape change was associated with the relative expansion of the paracone. Variation along PC2 (18%) consisted in the relative expansion of the talon with respect to the trigon.

### Covariation

**Figure 2:**
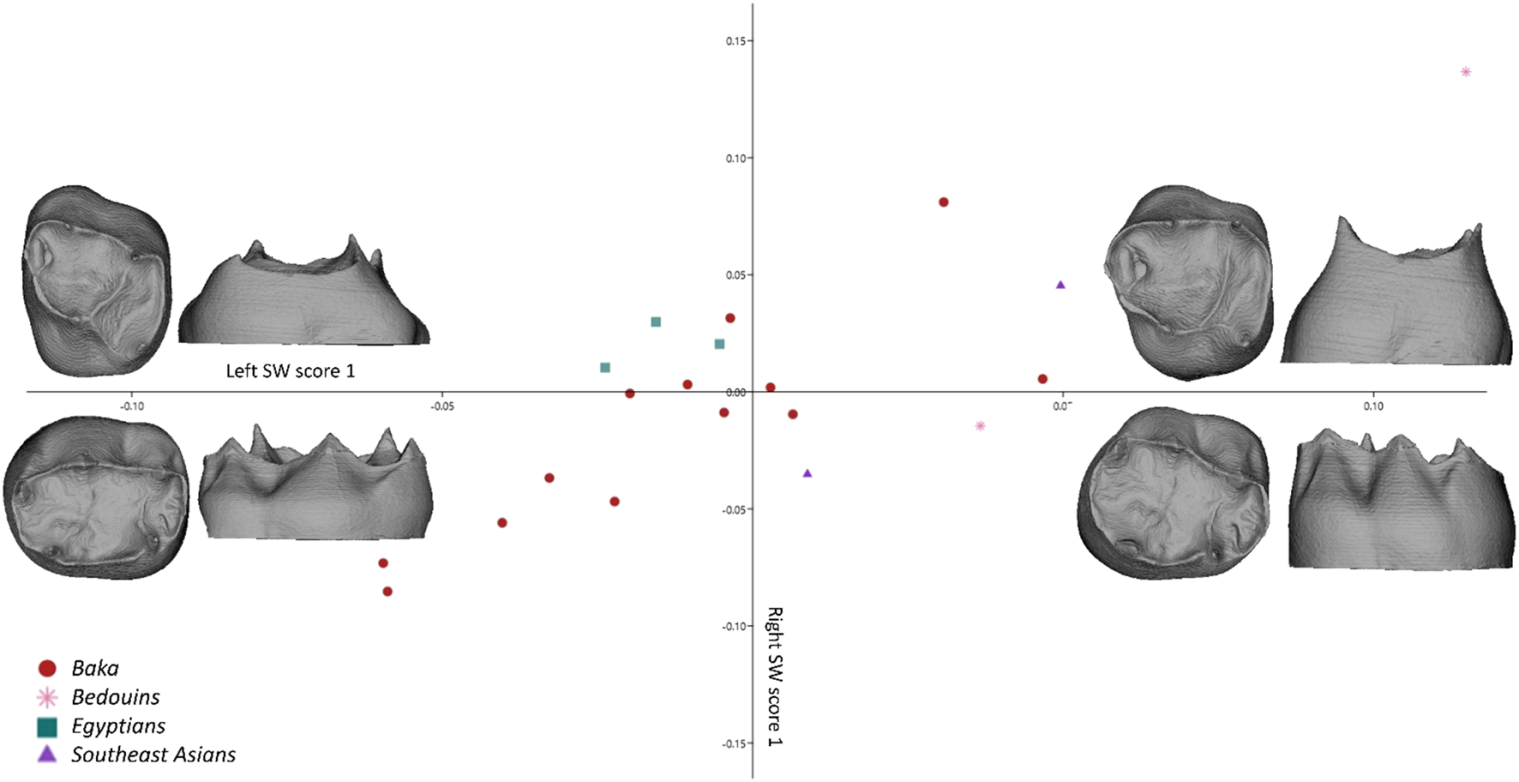
2B-PLS plot capturing the covariation between dentinal crowns (combining enamel-dentine junction and cervical outlines) of deciduous upper and lower second molars (dm2s; warpings at values ± 0.15). The upper and lower dm2 dyads vary from low-crowned with expanded opposing fossae to high-crowned with reduced opposing fossae. The various populations can be hardly distinguished, although the subsample sizes are too small to allow definite statements. Within the Baka sample the most extreme expression towards the low-crowned variation is observed.

The covariation between upper and lower dm2s could be assessed only for individuals represented by both tooth types (n = 20 of which 13 Baka). For the dentinal crown, the percentage of total squared covariance of udm2s and ldm2s for the Singular Warp scores 1 was 66% and the pairwise shape correlation between the antagonists was r1 = 0.82 (Table 3). Upper and lower dm2s clearly showed common trends of variation (Fig. 2), being either short-crowned with reduced central fossa (in udm2s) and distal fossa (in ldm2s), or taller with expanded opposing fossae. Moreover, lower dentinal crowns were associated with relatively higher horn tips than taller crowns. It was not possible to clearly separate the populations from each other based on the upper and lower dm2 dyads, although the Baka showed extreme expression towards low and elongated dentinal crowns. Within the same tooth type, the highest covariation coefficient was found between the ldm2s cervical and crown outlines (r1 = 0.81), while cervical and crown outlines in udm2s covaried the least (r1 = 0.74).

**Table 3:** Results of the 2B-PLS (single warp score 1) for the deciduous upper and lower second molars (udm2 and ldm2, respectively).

	Pairwise correlation (r1)	% of total covariance
	**udm2 dentinal crown**	**udm2 dentinal crown**
**ldm2 dentinal crown**	0.82	66
	**udm2 cervical outline**	**udm2 cervical outline**
**udm2 crown outline**	0.74	61
	**ldm2 cervical outline**	**ldm2 cervical outline**
**ldm2 crown outline**	0.81	51

The covariation between upper and lower dentinal crowns **(**combined enamel-dentine junction and cervical outline), and between cervical and crown outline in both udm2s and ldm2s was calculated.

### Size

**Figure 3:**
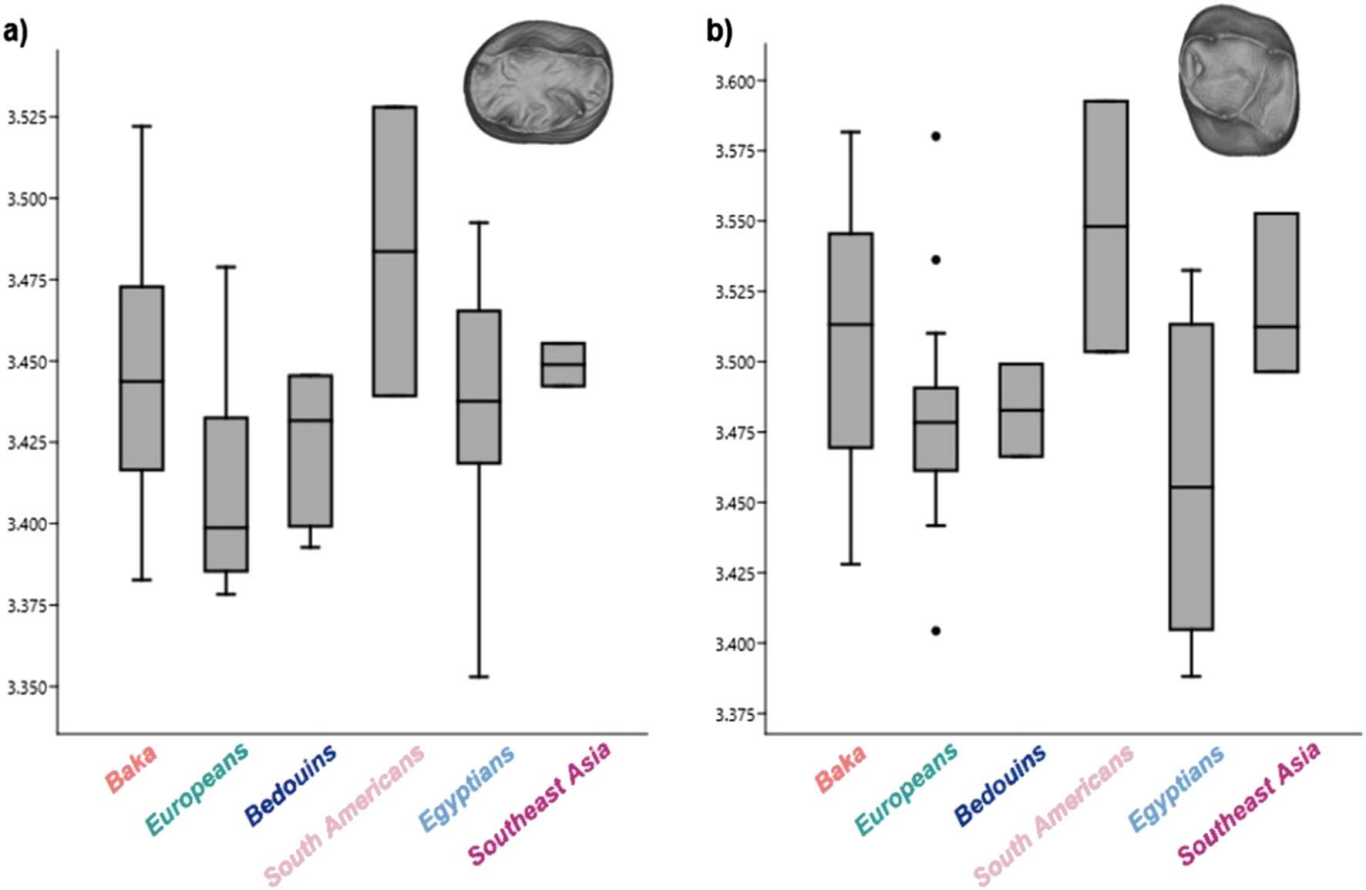
Boxplots of the natural logarithm of Centroid Sizes from the dentinal crown (combining enamel-dentine junction and cervical outline) in deciduous (**a**) lower and (**b**) upper second molars. South Americans and the Baka possess the largest deciduous second molars. The smallest specimens are within the Europeans and Egyptians.

The comparison of the Baka’s natural logarithm of Centroid Size (lnCS) for the dentinal crown with respect to the rest of the sample, showed that the Baka’s upper and lower dm2s were among the largest specimens within our sample following the South Americans (Fig. 3). With respect to the various populations, the Baka’s upper and lower dm2s differed significantly from Europeans, which similarly to the Egyptians were among the smallest specimens. Comparable results were obtained by observing the crown outline. The cervical and crown outlines of the Baka’s udm2s were significantly larger than the rest of the sample (Table 4). Interestingly, while the Baka possessed the largest udm2s cervical outline, the Egyptians showed the highest values for the ldm2s. However, the interpretation of the findings for the groups with the smallest sample size (i.e., Egyptians, South American, and Southeast Asian individuals) require caution.

**Table 4:** Results of the Mann–Whitney U test and Kruskal–Wallis test.

		**n (B/ n-B)**	**Z**	***p*-value**	**n (B/E)**	**Z**	***p*-value**	**Kruskal–Wallis test**	***p*-value**
**ldm2**	**Dentinal crown**	28/26	1.462	0.143	28/11	2.325	**0.020**	7.83	0.165
	**Cervical outline**	32/28	1.044	0.296	32/12	2.095	**0.036**	10.01	0.074
	**Crown outline**	32/27	1.072	0.283	32/11	2.157	**0.030**	6.78	0.237
**udm2**	**Dentinal crown**	22/33	1.898	0.057	22/22	2.325	**0.020**	10.97	0.052
	**Cervical outline**	23/37	3.740	**< 0.001**	23/22	3.485	**< 0.001**	18.45	**0.002**
	**Crown outline**	23/37	2.934	**0.003**	23/22	3.235	**0.001**	16.58	**0.005**

Differences in dental sizes (expressed by the logarithm of Centroid Sizes) for the deciduous upper and lower second molars (udm2 and ldm2, respectively) between the Baka and the rest of the comparative sample, between the Baka and the Europeans, and among all populations across the sample (B = Baka; n-B = non-Baka; E = Europeans; N = number of individuals).

The multivariate regression showed that only a low percentage of morphological variation of the dentinal crown could be explained by lnCS (ldm2: ~ 3%; udm2: ~ 9%). In ldm2s, we observed size-related shape variation mainly connected to the expansion and reduction of the distal and occlusal aspects, and the height of the dentinal crown. Larger ldm2s showed low dentinal crowns with expanded distal aspects and mesio-distally elongated occlusal aspects. This underlines the fact that the largest teeth (among which the Baka) are short, bucco-lingually narrow, and mesio-distally elongated, while the smallest (including Europeans) tended to be taller, bucco-lingually broader and mesio-distally shorter. Similarly, size-related shape changes in udm2s were associated to the height of the dentinal crown, the relative expansion of the metacone, and the bucco-lingual position of the hypocone. Larger udm2s (including the Baka) showed lower dentinal crowns with expanded metacone and buccally shifted hypocone, resulting in a squared crown as seen in occlusal view. Conversely, smaller udm2 specimens, including the Europeans, typically showed high crown, reduced metacone and lingually shifted hypocone.

The dm2 size differences between the Baka males and females were not statistically supported (see Supplementary Fig. S5 and Supplementary Table S2 online) based on the lnCS of the dentinal crowns or crown outlines. These analyses could not be carried out on the whole sample due to the lack of necessary meta-data.

### Non-metric traits

The prevalence of the hypocone and Carabelli’s cusp for the udm2s, and entoconulid and metaconulid for the ldm2s are presented in Table 5. The high degree of occlusal wear along with the resorption of the roots in the Baka specimens hampered the observation of further discrete traits. The hypocone was present in each of the udm2 specimens in our sample, and was the most variable trait although it was highly expressed in most of the individuals (grade 4 to 6)^24^. The Baka showed a significantly higher frequency of a massive hypocone manifestation (96%) in comparison to the rest of the sample (Chi^2^ = 11.7; *P* = 0.001). Most specimens, however, showed a medium grade of expression (3–4). In our sample, we found no or slight expression of Carabelli’s cusp, of the entoconulid, and the metaconulid, with no significant differences between populations.

**Table 5:** Prevalence and degree of expression of the deciduous upper and lower second molars’ (udm2 and ldm2, respectively) discrete dental traits, observed on the outer enamel surface.

		Prevalence (%)						
ldm2	Trait expression	Baka (n = 32)	Europe (n = 11)	Southeast Asia (n = 3)	Egypt (n = 7)	South America (n = 2)	Bedouins (n = 4)	Complete sample
Entoconulid	0–2	88	100	100	100	100	75	34
	3–5	12	–	–	–	–	25	
Metaconulid	0,1,1A	75	82	67	72	50	75	54
	2–4	25	18	33	28	50	25	

udm2	Trait expression	Baka (n = 23)	Europe (n = 22)	Southeast Asia (n = 5)	Egypt (n = 6)	South America (n = 2)	Bedouins (n = 2)	Complete sample
Hypocone	0–3	4	50	40	33	100	–	100
	4–6	96	50	60	67	–	100	
Carabelli’s cusp	0–3	83	95	100	83	100	100	60
	4–7	17	5	–	17	–	–	

*n* number of individuals.

## Discussion

The Baka Pygmies from south-eastern Cameroon, western Central African Republic, and northern Congo^5–8,10,18^ are characterized by their peculiar life history and growth pattern with respect to other Pygmy and non-Pygmy populations. They are also known for their large permanent molars and early eruption times of the permanent teeth. However, in terms of dental morphology, we found that their dm2s are indistinguishable from other non-Pygmy populations. In fact, we observed a great overlap between populations in our ldm2 sample, which hampered the possibility to distinguish the diverse geographical groups based only on their tooth shape. Similar findings were obtained for permanent upper and lower premolars^25,26^, and first and second molars^27,28^ from non-Pygmy populations.

Based on dental crown diameters, most of the variation occurs within rather than between populations^29,30^. The high degree of shape variation observed in the Baka’s ldm2s is compatible with previous observations on Sub-Saharan populations, which have been proven highly variable in terms of both dental^29^ and cranial dimensions^31,32^. Surprisingly, the extended shape distribution of the Baka’s lower dm2s is not paralleled by the upper dm2s. Interestingly, the pattern of variation in European dm2s follows the opposite trend, with less variable lower dm2s and highly variable upper dm2s. This finding is consistent with our knowledge of the European permanent upper molars showing a high degree of hypocone and metacone reduction^28^.

Given the morphological resemblances of the dm2s to first molars (M1s)^33–37^, we draw comparisons of our results with those previously published on M1s^28,38,39^. Distal and lingual aspects appear to be the most variable regions in both upper and lower dm2s. This finding is in agreement with Halász’s^28^ research on M1s from various populations, and with Polychronis et al.^39^, who studied M1s from Greek individuals. There seems to be a clear trend that points to an increased shape variation in the distal aspect of the crown. The pattern of shape covariation observed between upper and lower dm2s, from tall-crowned with expanded opposing fossae to short-crowned with reduced opposing fossae, was also observed in upper and lower M1s^28^. High pairwise correlation between opposing teeth developing in different jaws can be interpreted as a strong hint to tight genetic control over the dental development and is explained by the fact that antagonists have to occlude optimally to facilitate effective mastication. Likely, these functional constraints concern all posterior teeth, in fact similar results were achieved for dyads of other dental types^26,28^.

In terms of non-metric traits, our results for the hypocone contrasted the findings of Edgar and Lease^33^ who observed a high percentage of reduced hypocone expression in deciduous molars in general. Instead, we found expression of larger hypocones in the Baka than in Europeans, as Lease^40^ found between African Americans and Europeans. Today it is known that the manifestation of Carabelli’s cusp is equally common in all world populations^41^, which was confirmed by our findings. However differently from Halász^28^ who observed a prevalence of 100% in African and Near Eastern M1s, and in accordance to Scott^42^ we found that most of the specimens lacked Carabelli’s cusp. According to Harris^43^ the grade of expression of Carabelli’s cusp in M1s and the tooth size are connected, with larger teeth showing this trait more often. We cannot support this result, since we did not find any significant differences in Carabelli’s cusp manifestation between the populations in spite of the significant size differences. Likewise, the various populations used in our sample did not differ in terms of entoconulid and metaconulid frequency or degree of expression.

Variation in dental size of the Pygmies’ permanent dentition in comparison to non-Pygmy populations was assessed previously for various samples testing different hypotheses^18–20,44^. The general findings include the tendency of sub-Saharan African populations to show larger permanent teeth than Asians or Europeans^30^, which was confirmed by our results. According to Hanihara and Ishida^30^, the modern aboriginal Australians show the largest dental diameters, followed by a group of average-sized dentitions of sub-Saharan Africans, Native Americans, and Southeast Asians. Europeans and populations of the Near East showed the smallest dentition. We observed the smallest dm2s sizes in Europeans and Bedouins, followed by Egyptians, Southeast Asians, and sub-Saharan Baka Pygmies. South Americans showed the largest dm2 crowns. It is worth nothing that our results use lnCS rather than linear distances, a different measure of size, depending also on the landmark configuration^26^. The dental size of the Baka and other Pygmies was studied also in relation to their body size and developmental patterns^19,20^. The results for the Baka’s dm2s match previous findings on permanent molars which are larger than in any other non-Pygmy population^20^, including the Bantu^18^. However, the Baka’s dm2s do not show sex-related size variation that was previously reported for the permanent molars^18^.

Early dental eruption timing has not only been found in the Baka’s dm2s^3^ but also in other sub-Saharan populations’ permanent^45–48^ and deciduous teeth^49^. According to Lam et al. (2015)^50^, the earlier onset of tooth eruption can be associated with postnatal factors such as an increased rate of weight gain during the first three months of life. It is worth noting that, contrary to the Sua Pygmies, the Baka’s weight gain during the first years of infancy does not differ from that of the Bantu^3^. However, considering that the time of dm2 formation partially overlaps with the time of growth decrease in the Baka, and the dm2s’ size is not negatively affected by the decrease in body growth, we can assume no or only a very low correlation between odontogenesis and body growth in the Baka. Romero et al.^18^ and Ramirez Rozzi^19^ underline this finding stating that short stature is likely an adaptation not related to dental shape and size variation, and is genetically inherited by the Baka’s ancestors^51^. Furthermore, no clear genetic pathway affecting both the teeth and the somatic growth that could explain the phenomena we observed in the Baka has yet been found^19^. Earlier tooth eruption has been associated with a lower degree of root growth^52^, although root development and morphology in the Baka have not been studied yet, therefore the mechanisms prompting an early eruption of their teeth remain unknown.

In conclusion, despite the Baka’s long-lasting geographical isolation^3,51^, very low levels of genetic admixture with non-Pygmy neighboring populations^51,53^, specific growth pattern, and dental eruption timing, we did not find a morphological difference distinguishing the Baka from other world populations based only on their dental morphology. As expected, we also did not observe any significant shape differences between sexes. Yet, the Baka’s ldm2s can be particularly narrow and elongated with respect to the rest of the sample analyzed, and udm2s also clearly exceed the range of low and squared-crown variation in the sample, despite the fact that they are less variable than the ldm2s. Outstandingly, differential patterns of variation of udm2s and ldm2s in Central Europeans and the Baka might reflect their different population history reaching back to the migration of the European ancestors out of Africa^17^, a topic that deserves further attention. Still, assigning an individual tooth to a certain population based on its shape alone remains impossible.

## Methods

### On the usage of the term ‘pygmy’

In spite of the fact that term pygmy has been used by some as pejorative, its etymology is not dubious nor derogatory, deriving from the Greek pygmē ‘a cubit’, the measure of length from the elbow to the extreme of the middle finger (for an historical account of the usage of the term pygmy see Ballabriga, 1981^54^; Bahuchet, 1993^55^; Ramirez Rozzi, 2015^56^; 2021^57^). From a scientific standpoint, population genetics has shown that all human groups living in the African Equatorial forest usually referred to as Pygmies, shared a common ancestor which split from non-Pygmy groups around 65–50,000 years ago^51,53^: The term pygmy identifies groups of humans based on their geographic distribution, morphology, adaptation to the environment, growth pattern, and cultural and social traits: the Pygmies have a distinct population history which deserves to be acknowledged. In fact, adopting the term ‘Pygmy’, the Baka distinguish themselves from the Nzime, a group of Bantu ethnicity living in the surroundings of the Bosquet area. Some among the detractors of the term Pygmy have suggested the use of the term 'twa' (meaning ‘dwarf’) as an alternative. However, the Baka strongly oppose to defining themselves as such since non-Pygmy neighbor populations use it with disparaging intent. Others have proposed 'hunters-gatherers', but this term accounts only for the subsistence strategy thereby clouding all of the other distinctive traits of the Pygmies. The Pygmies have faced centuries of oppression which have led them to hide their identity to escape persecution^58^. Thus, referring to the Baka as Pygmies, is not only proper in scientific and anthropological terms, but is also ethically correct.

### Sample

Our sample consists of 60 upper and 59 lower modern human deciduous second molars (Table 1). Of these, 55 teeth, including 23 udm2s and 32 ldm2s, are from 39 Baka individuals. The Baka were collected by F.V.R.R. after the Baka children naturally shed and donated them with the consent of their families. The rest of our sample was composed of individuals from different geographical regions, including Europe (udm2 = 22; ldm2 = 11), Africa (udm2 = 6; ldm2 = 7), Asia (udm2 = 5; ldm2 = 3), the Near East (udm2 = 2; ldm2 = 4), and South America (udm2 = 2; ldm2 = 2), which is quite a large sample considering the paucity of infant and juvenile specimens in osteological collections. More so, the meta-data for the comparative sample is often incomplete since the individuals come from archaeological collections (Table 1). Other limitations of the sample size are the wear stage and state of preservation of the teeth. Dental specimens showing a moderate abrasion of the horn tips (stages 3 and 4) were virtually reconstructed (see *µCT acquisition and data segmentation* below) before data collection. To achieve a successful virtual reconstruction of the EDJ surface, wear cannot exceed stage 4^23^, thus specimens showing higher degree of wear or with extensive decay in the occlusal area were either excluded from the sample or used only for the analyses of the cervical and crown outlines (see below). The heavy wear, the poor state of preservation, and occasional dental treatment made a large number of the Baka dental collection unusable for our study. In fact, the Baka’s traditional dental treatments are quite invasive and entail the drilling of large cavities later filled with natural substances^9^. The Baka teeth were naturally shed and thus were in function approximately until the age of 11 which explains their advanced degree of wear. In some cases, the molars were kept by the Baka children or their families until the next visit of F.V.R.R., which likely led to the poor state of preservation of the teeth at the moment of image data acquisition (i.e., for the presence of numerous and deep cracks).

### µCT acquisition and data segmentation

The dental datasets were imaged at four different facilities. The Baka teeth were scanned mainly at the Plateforme Imageries du vivant, Université de Paris, using a Micro CT-scanner PerkinElmer, Quantum FX (voxel size 20-40/30-59/10-20 µm, 90 kV, 16 mA) and at the Hard Tissue Research Unit, College of Dentistry (NYU), with a SCANCO Scantron 40 Micro-CT scanner (voxel size 12 µm, 70 kV, 275 mA, 200 ms). The comparative sample was scanned at the Centre de Microtomographie of the Université de Poitiers, with a VISCOM X8050-16 (voxel size 24–60 µm, 95–110 kV, 0.5 mA), and at the Vienna Micro-CT Lab, Austria, with a VISCOM X8060 NDT scanner (voxel size 21–60 μm, 110–140 kV, 280–410 mA, 1400–2000 ms, 0.75 mm copper filter). X-ray images were taken from 1440 different angles. Using filtered back-projection in VISCOM XVR-CT 1.07 software, these data were reconstructed as 3D volumes with a color depth of 16,384 grey values.

The µCT data were then imported into Amira software (www.fei.com) and virtually segmented to separate the enamel from the dentine, pulp, and the surrounding material (i.e., air, alveolar bone). In case of a slight abrasion of the dentinal horn tips, the specimens were virtually reconstructed by using the “brush” tool and extending the contours of the existing dentine into the empty area. In case both left and right dm2s were available from one individual, we preferred the left ones among our whole sample. However, if better preserved, we used the right tooth after virtual mirroring to the left side. Since there is no scientific evidence indicating the existence of directional asymmetry in human dentition, we assume that the choice of left teeth should not affect the results of this study^59–61^.

### Reorientation and outline collection

After segmentation, the surface models of the crowns were consistently reoriented in Geomagic Design X 64 (www.3dsystems.com) following an established protocol^62–64^. The crown and cervical outlines were collected from the reoriented surface models and projected onto the cervical plane. Afterwards, the outlines were split into 24 segments by as many equiangular radial vectors originating from the centroid of the outline area, using Rhinoceros 6 (www.rhino3d.com). Twenty-four pseudo-landmarks were placed at the point of intersection of the radii and the outline.

### Landmark collection on the enamel-dentine junction

To collect the landmarks on the EDJ, we followed established protocols^62–64^. For the ldm2s, we placed a total of eight landmarks (LM) on the five main horn tips and three at the deepest points between metaconid and entoconid, protoconid and hypoconid, and between hypoconid and hypoconulid. Afterwards, 23 curve semilandmarks (sLM) were placed to represent the EDJ marginal edge. To ensure homology, we traced the EDJ occlusal edge by creating a spline curve ignoring all the accessory cusps. For the udm2s, seven LMs were placed on the four horn tips, and on the deepest points of the central fovea and distal fossa, and the deepest point of the lingual marginal ridge of the hypocone. The EDJ marginal edge was resampled by 47 sLMs. The LM collection was carried out in the EVAN Toolbox 1.75 (www.evan-society.org), which uses the bending energy technique for sliding the sLM^65–67^.

### Geometric Morphometric analysis

The geometric morphometric analyses were performed using the EVAN Toolbox separately for each set of landmarks, resulting in four different analyses per tooth type, namely: (1) cervical outlines; (2) crown outlines; (3) EDJ and (4) dentinal crown, combining the landmark configuration of the EDJ with the cervical outline. First, the landmark configurations had to be normalized via General Procrustes Analysis (GPA)^65,68,69^. The translation and rotation of the landmark sets were not necessary for the outline configurations since they were manually aligned. We run the principal component analysis (PCA) on the Procrustes shape coordinates and visualized the shape changes along the principal components by means of warpings, using the Thin-Plate Spline technique^70,71^. The analyses of the size were carried out using the natural logarithm of Centroid Size (lnCS). The lnCS of the combined EDJ and cervical outline is an accurate measure of the dentinal crown size since it embeds information about the crown’s height, too. We explored shape covariation between udm2s and ldm2s as well as between different features of the same dental types by means of the 2-block partial least squares analysis (2B-PLS). Using R Studio (www.r-project.org), we performed the analysis of variance on Procrustes shape coordinates of larger subsamples, i.e., the Baka and the Europeans, to assess their degree of variation in the morphological expression of udm2s and ldm2s. In addition, the group mean differences between male and female Baka dentinal crowns were assessed with a permutation test of the Procrustes distances (10,000 random permutations) using a R script written for this purpose. A Mann–Whitney U test was used to test significance in size differences between the Baka and the Europeans, and between the Baka and the rest of the sample. Another one was performed to the sex-related size differences within the Baka. This analysis could not be carried out on the other populations owing to the lack of associated meta-data. These tests have been carried out using PAST 4.03 (www.softpedia.com), as well as the Kruskal–Wallis test used to assess the size differences on populational level across the whole sample. Moreover, we performed a multivariate regression to analyze size-related shape variation of the dentinal crown.

### Non-metric traits

The non-metric traits were evaluated based on the Arizona State University Dental Anthropology System (ASUDAS)^24,72^. Because of the high degree of wear of the Baka sample and root resorption, we focused on four among the most informative dental traits visible on the outer enamel surface, possibly reflecting neutral genetic variation^21,73^.

1. Hypocone
  The hypocone is the fourth cusp of the upper molars that forms a separate region of the occlusal aspect, the trigon. Its manifestation varies from grade 0 to 6^24^. In this study, we used dichotomous classes to represent the hypocone degree of expression: none/light (0–3) and moderate/heavy (4–6).
2. Carabelli’s cusp
  The Carabelli’s cusp is an upper molar’s accessory cusp occurring on the protocone (mesio-lingual cusp). This trait has been used as a diagnostic trait for European populations^42^, however, according to more recent studies, no differences in prevalence and expression between populations are expected^24^. According to ASUDAS, the expression of the cusp of Carabelli varies between grades 0 to 7, but for this study, we used two categories: none/light (0–4) and moderate/heavy (5–7).
3. Entoconulid
  The entoconulid, or cusp 6, can be found in the distal area of the occlusal aspect of lower molars between the hypoconulid and the entoconid. The manifestation of the entoconulid can be expressed between grades 0 to 5, which are grouped into none/light (0–2) and moderate/heavy (3–5) in our study.
4. Metaconulid
  The metaconulid, or cusp 7, can be found on the lingual aspect of the lower molars between the entoconid and metaconid. Its manifestation can be expressed with 6 grades: 0, 1, 1A, 2, 3, and 4. We dichotomized the scoring values as none/light (0 to 1A) and moderate/heavy (2 to 4).

The prevalence of the non-metric traits was analyzed by Chí^2^ test using SPSS (www.ibm.com).

### Ethical statement

We did not use any physically invasive or destructive methods in our study. Our sample included only teeth obtained from archaeological collections or donated by the Baka children after they naturally shed them, and thus were not extracted. The Baka teeth were collected during field works carried out as part of an international agreement between the French National Research Institute for Development (IRD) and the Ministry of Scientific Research and Technology of Cameroon (Accord-cadre de Coopération en matière de Recherche Scientifique et Technique, 2004). Accordingly, informed consent was obtained from all participants and from both parents of any participants aged under 18.

## Supplementary Information

Supplementary Information.

## Data Availability

All data generated or analyzed during this study are either included in this published article (and its Supplementary Information file) or available from the corresponding author on reasonable request.
