# Supplementary material for: Morphological variation of the deciduous second molars in the Baka Pygmies
Source: Sci Rep. 2021 Aug 13;11:16480. doi: 10.1038/s41598-021-95524-3 (PMC8363745; doi:10.1038/s41598-021-95524-3)
Supplement: Supplementary file 1 — Supplementary Information. [file 41598_2021_95524_MOESM1_ESM.docx]

**Morphological variation of the deciduous second molars in the Baka Pygmies**

**Petra G. Šimková^1^, Gerhard W. Weber^1,2^, Fernando V. Ramirez-Rozzi^3,4^, Lotfi Slimani^5^, Jérémy Sadoine^5^, Cinzia Fornai^1,6,7^**

^1^Department of Evolutionary Anthropology, University of Vienna, Austria. ^2^Core Facility for Micro-Computed Tomography, University of Vienna, Austria. ^3^UMR7206 Ecoanthropologie, MNHN, CNRS, UP, Musée de l’Homme, Paris, France. ^4^EA 2496 Pathologies, Imagerie et biothérapies oro-faciales, Université Paris Descartes, Montrouge, France. ^5^UR2496 - Plateforme Imageries du Vivant, Université de Paris, Montrouge, France. ^6^Institute of Evolutionary Medicine, University of Zurich, Switzerland. ^7^Vienna School of Interdisciplinary Dentistry, Klosterneuburg, Austria.

**
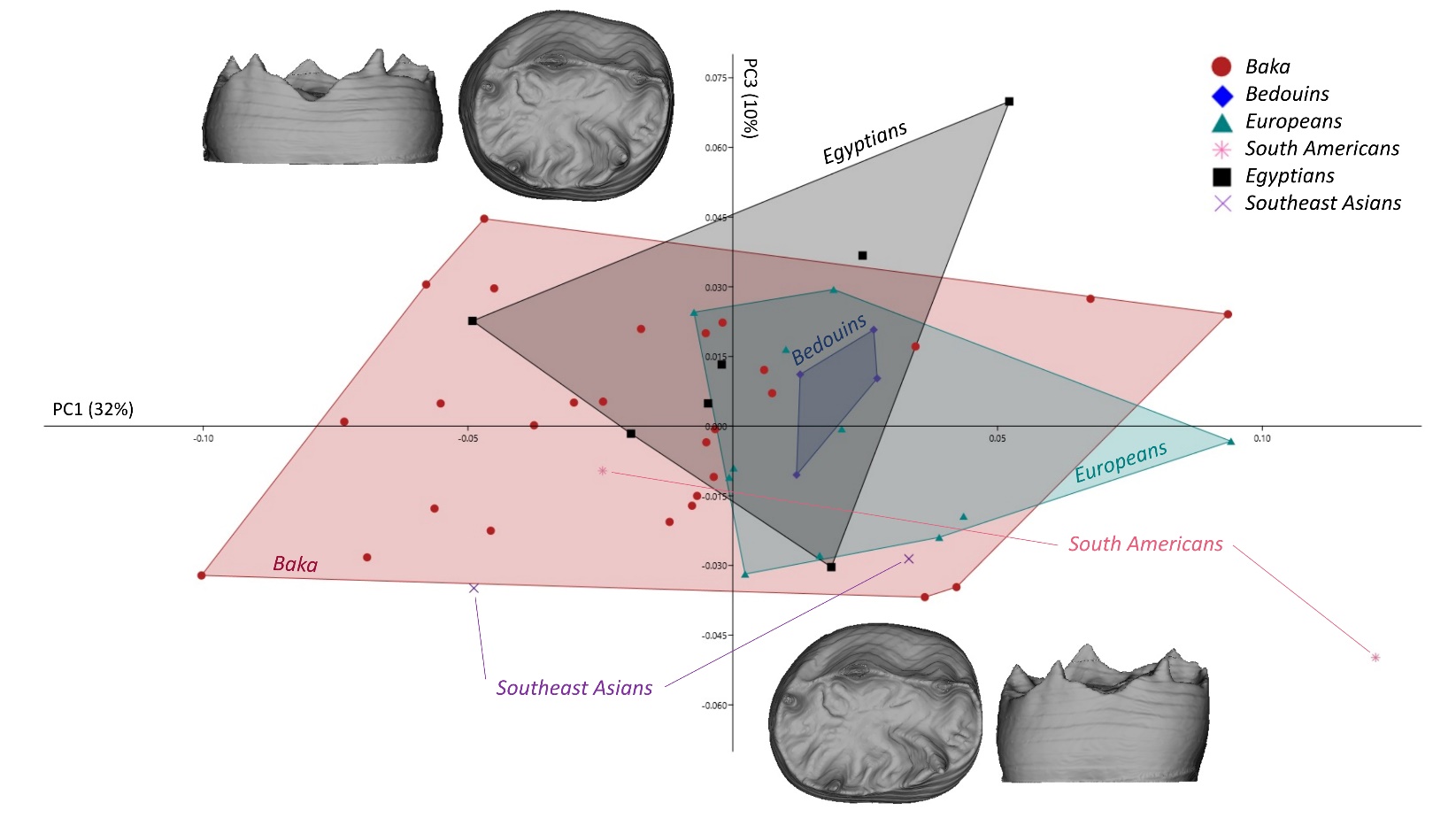
**

**Supplementary Figure S1 PC1 – PC3 plot for the deciduous lower second molars in shape space for the dentinal crown, combining enamel-dentine junction and cervical outline**. Along PC3 (explaining 10% of the total variance), the human groups overlap extensively. Shape changes are driven by the relative size of the tooth base in relation to the occlusal aspect, as well as changes in the crown height that increases with decreased relative size of the base (warpings at values ± 0.07). For the description of the results along PC1, see Fig. 1a in the main text.


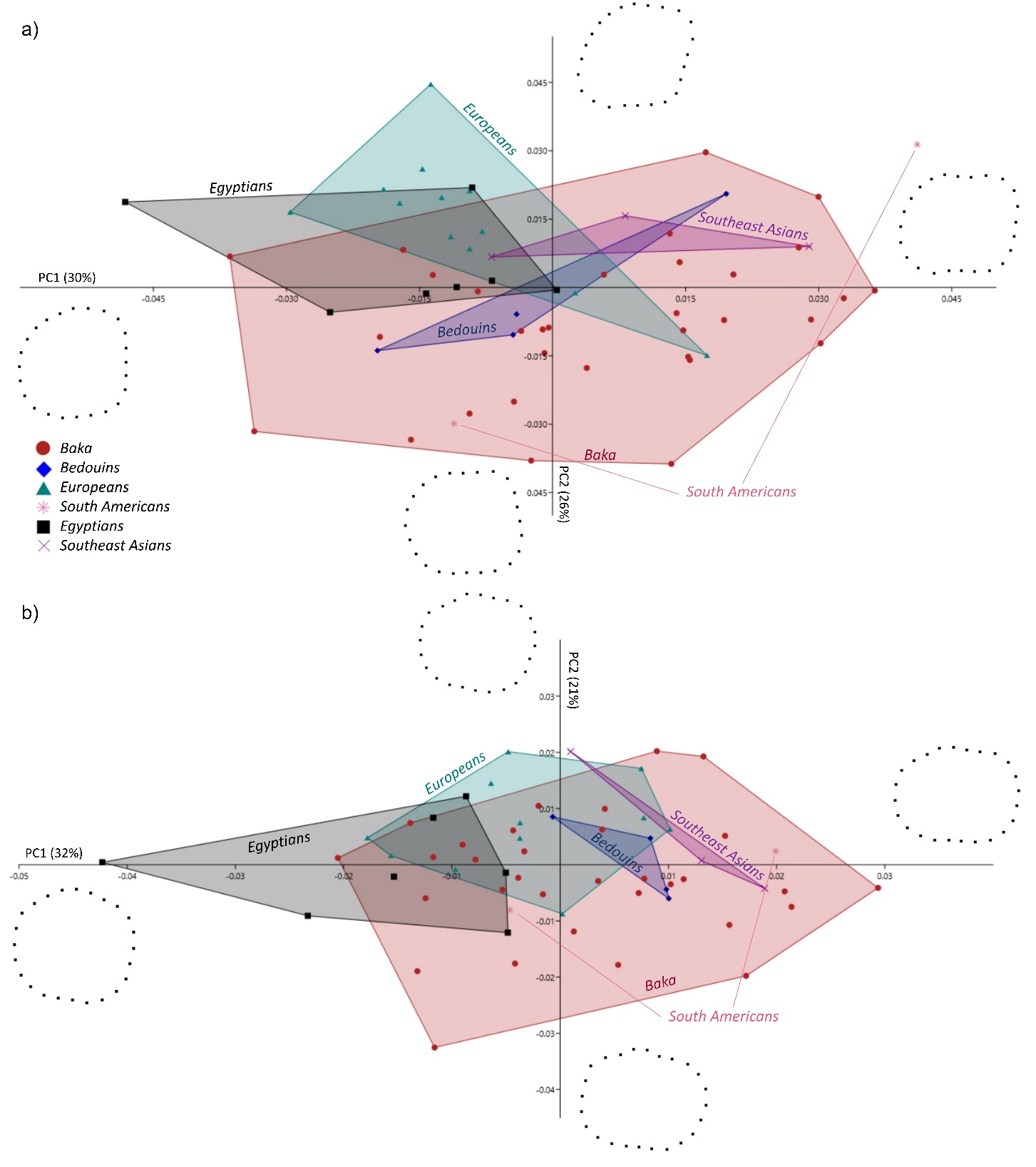


**Supplementary Figure S2 PCA plots for the deciduous lower second molars’ dental outlines.** a) PC1 - PC2 plot in shape space for the cervical outline (warpings at values ± 0.05). The Baka exceed the range of variation of the rest of the sample, for the particularly pronounced hourglass-like shaped cervical outlines; b) PC1 - PC2 in shape space for the crown outline (warpings at values ± 0.05). The Baka show an extreme variation expression in elongated and bucco-lingually constricted crown outlines.


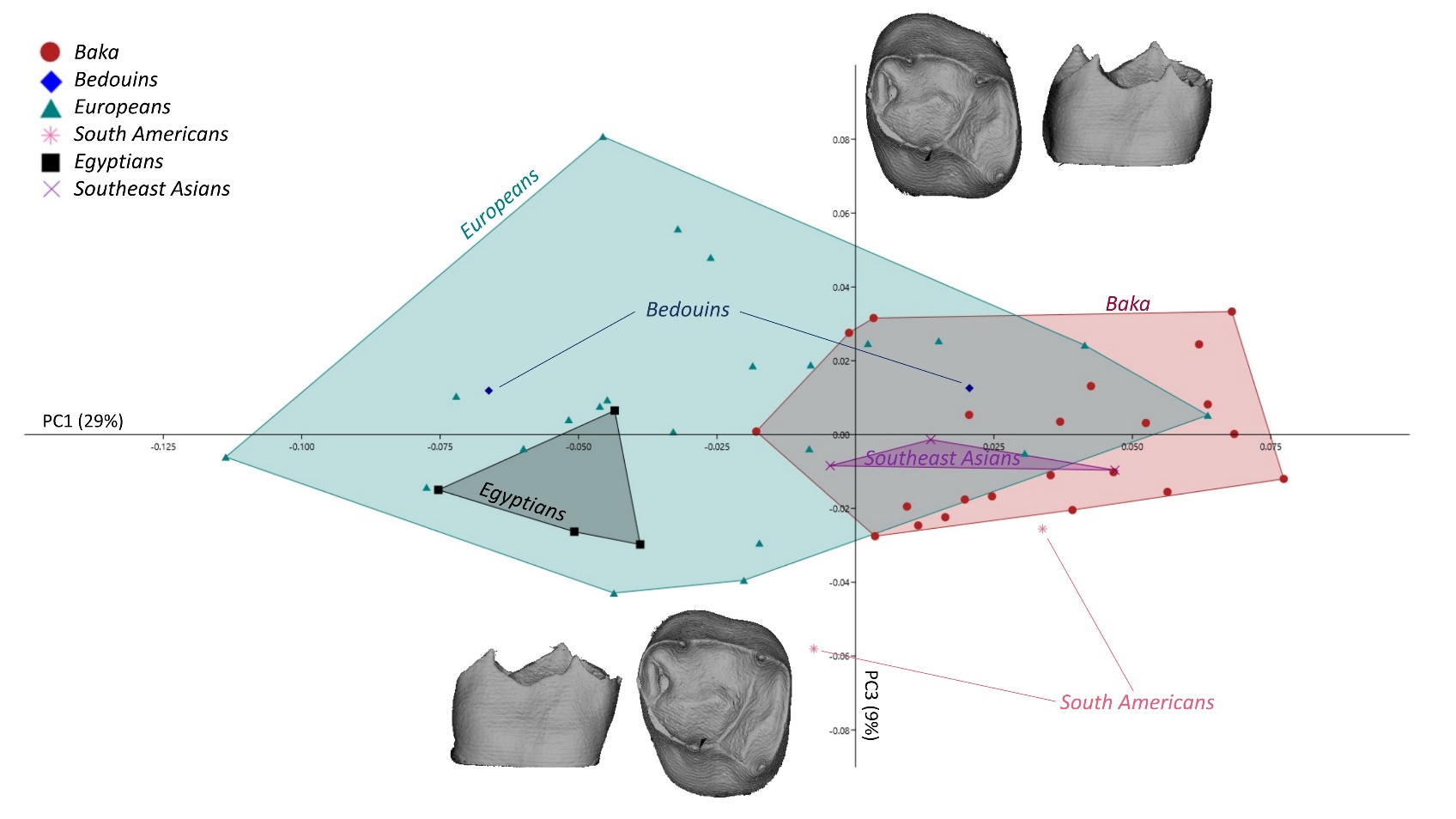


**Supplementary Figure S3 PC1 – PC3 plot for the deciduous upper second molars in shape space for the dentinal crown, combining enamel-dentine junction and cervical outline**. Along PC3 (explaining 9% of the total variance), the variation is driven mainly by the relative expansion of the trigon with respect to the talon. The reduction of the trigon is associated with observed metacone reduction. The Europeans exceed the range of variation of the rest of the sample in both directions, while the Baka are much less variable and centered around the mean values (warpings at values ± 0.085). The two South American specimens possess particularly reduced talons. For the description of the results for PC1 see Fig. 1b in the main text.


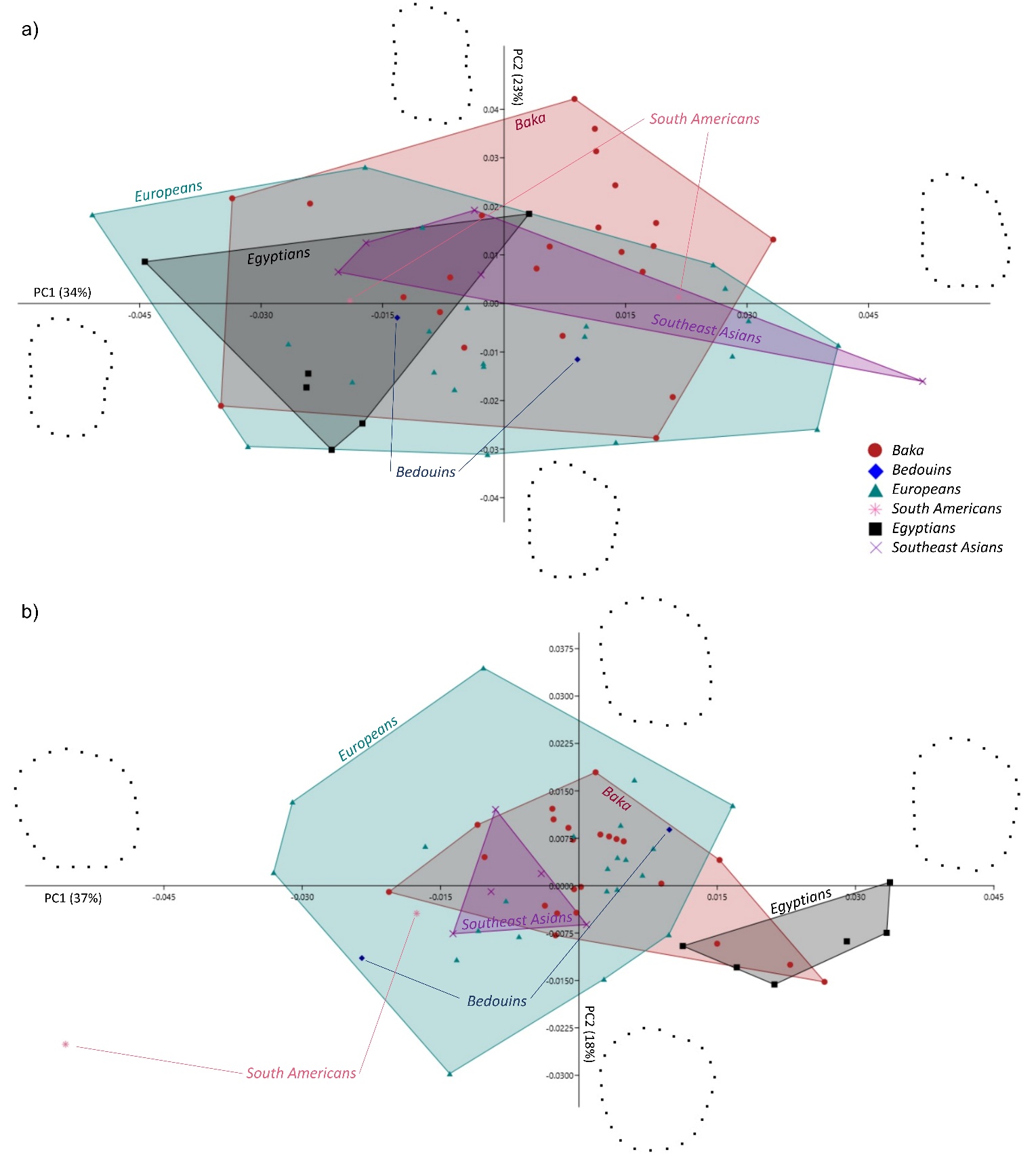


**Supplementary Figure S4 PCA plots for the deciduous upper second molars’ dental outlines** a) PC1 - PC2 plot in shape space for the cervical outline (warpings at values ± 0.05). The human groups overlap extensively; b) PC1 - PC2 plot in shape space for the crown outline (warpings at values ± 0.04). The Europeans are more variable than the Baka and exceed the range of variance of the sample along PC2.


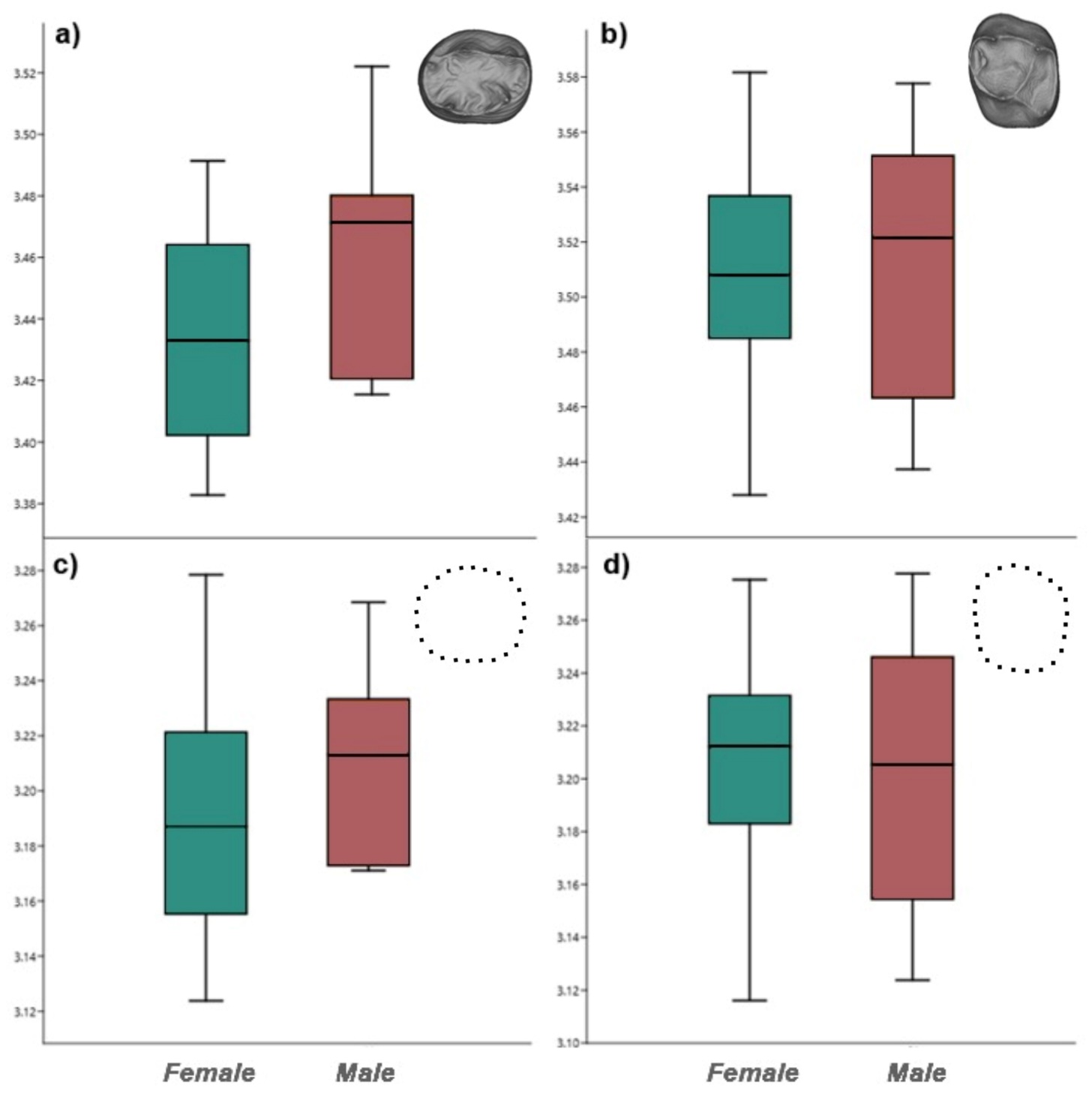


**Supplementary Figure S5 Boxplots of the natural logarithm of Centroid Sizes for the Baka’s male and female deciduous second molars (dm2s).** The comparison of the lnCS distributions for the **a)** lower and **b)** upper dentinal crowns, and **c)** lower and **d)** upper crown outlines, do not reflect marked sexual differences, although a trend for larger male lower dm2s is noticeable.

| **Supplementary Table S1 Variance of the Procrustes shape coordinates for the Baka’s and Europeans’ dentinal crowns** (combining enamel-dentine junction and cervical outline) of the deciduous upper and lower second molars (udm2s and ldm2s, respectively). | | |
| --- | --- | --- |
|  | **The Baka** | **Europeans** |
| **ldm2** | 0.006 | 0.005 |
| **udm2** | 0.005 | 0.007 |

| **Supplementary Table S2 Results of the Mann-Whitney U test on the Baka’s male and female logarithm of Centroid Sizes.** Differences in dental sizes between the Baka’s male and female dentinal crowns and crown outlines (f = female; m = male; N = number of individuals) are not statistically significant. | | | | |
| --- | --- | --- | --- | --- |
|  | **ldm2** | | **udm2** | |
|  | **Dentinal crown** | **Crown**  **outline** | **Dentinal crown** | **Crown**  **outline** |
| **N (f/m)** | 17/11 | 18/14 | 9/13 | 9/14 |
| **Z** | 1.740 | 1.234 | 0.133 | 0.094 |
| ***p*-value** | 0.081 | 0.216 | 0.893 | 0.924 |
